# Indicators of psychiatric institutionalization in Southeast Asia between 1990 and 2024

**DOI:** 10.1016/j.lansea.2025.100690

**Published:** 2025-11-01

**Authors:** Adrian P. Mundt, S.M. Yasir Arafat, Chencho Dorji, Avinash Desousa, Rakesh K. Chadda, Guru S. Gowda, Cristian Orus, Muralidharan Kesavan, Athifa Ibrahim, Nagendra P. Luitel, Harischandra Gambheera, Phunnapa Kittirattanapaiboon, Stefan Priebe, Enzo Rozas-Serri

**Affiliations:** a Centro de Investigación Biomédica, Facultad de Medicina, Universidad Diego Portales, Santiago, Chile; b Departamento de Psiquiatría y Salud Mental, Facultad de Medicina, Hospital Clínico Universidad de Chile, Santiago, Chile; c Department of Psychiatry, Enam Medical College and Hospital, Dhaka, Bangladesh; d Jigme Dorji Wangchuk National Referral Hospital, Thimphu, Bhutan; e Department of Psychiatry, Lokmanya Tilak Municipal Medical College, Mumbai, India; f Department of Psychiatry and National Drug Dependence Treatment Centre, All India Institute of Medical Sciences, New Delhi, India; g Department of Psychiatry, National Institute of Mental Health and Neuro Sciences (NIMHANS), Hosur Road, Bengaluru, India; h Managing Partner, Epic Consulting LLP, Malé, Maldives; i Transcultural Psychosocial Organization Nepal, Baluwatar, Kathmandu, Nepal; j Consultant Psychiatrist – Retired from the National Institute of Mental Health, Angoda, Sri Lanka; k Director of Bureau of Mental Health Academic Affairs, Department of Mental Health, Ministry of Public Health, Nonthaburi, Thailand; l Centre for Psychosocial Medicine, University of Hamburg, Germany; m Departamento de Neurología y Psiquiatría, Clínica Alemana de Santiago, Facultad de Medicina Clínica Alemana, Universidad del Desarrollo, Santiago, Chile

**Keywords:** Psychiatric inpatient beds, Institutionalization, Prison population, Southeast Asian region, Prison population/psychiatric beds ratio

## Abstract

**Background:**

This study aimed to assess indicators of psychiatric institutionalization and their changes over time across the Southeast Asia Region (SEAR).

**Methods:**

We collected numbers of psychiatric beds, specialized forensic psychiatric beds, beds in residential facilities for people living with chronic mental illness and prison populations in the 11 SEAR member states between 1990 and 2024 using primary and secondary data sources. We calculated median rates per 100,000 population, as well as percent changes of the median rates between the first and last available data points. We also compared findings in SEAR with OECD countries.

**Findings:**

Psychiatric bed numbers and prison population were available from 10 countries. Bed prevalence increased in seven countries, but decreased in three. The median psychiatric bed prevalence was 1.5 at the first and 2.9 per 100,000 population at the last data point (+94%). The prison population increased in nine countries, with the median rate changing from 60 to 123 (+106%). Data on specialized forensic psychiatric beds were available from seven countries, with a range of 0.0–0.5 beds at the last data point. Based on data from five countries, the median prevalence of beds in residential facilities increased from 1.3 to 2.3. Psychiatric bed prevalence was on average about 5% of that in the OECD, while prison population rates were similar to those in OECD countries.

**Interpretation:**

Psychiatric bed provision in the SEAR is among the lowest worldwide. In contrast, incarceration rates are similar to those in high-income regions. Most countries have increased the prevalence of psychiatric beds over the past three decades, but they remain scarce, and further investments need consideration.

**Funding:**

10.13039/501100020884Agencia Nacional de Investigación y Desarrollo (ANID), Chile.

**Research in context**

**Evidence before this study**

Numbers of psychiatric beds are well documented and quantified for a range of high-income countries. However, research from the Southeast Asia Region (SEAR) is scarce. For this study, we reviewed data from the WHO Mental Health Atlas, World Bank data, PubMed, and Google Scholar (1990–2024). This revealed limited mental health resources in SEAR, with a high burden of mental illness and a treatment gap, potentially leading to criminal justice involvement for those with severe mental health conditions. We did not find studies that examined psychiatric bed prevalence and prison population rates longitudinally over time in the SEAR.

**Added value of this study**

This is an international collaborative work, involving authors from six LMICs in the SEAR, thereby providing a diverse range of views. The study compiled and quantified data on general psychiatric, forensic and residential psychiatric beds and incarceration rates across ten SEAR countries. It provides a comprehensive, longitudinal analysis (1990–2024), offering a regional perspective and a view on long-term trends, and facilitating cross-country comparisons. Most of these data were previously unpublished and unavailable in the public domain. The study uses an indicator—the ratio between the prison population and available psychiatric beds—to assess the balance in developments between punitive and therapeutic forms of institutionalization or care. A disproportional lack of available psychiatric beds compared to incarceration capacities was identified in the region.

**Implications of all the available evidence**

While psychiatric bed provision increased in the SEAR, it remained very low as compared to global standards and OECD nations. Further investments in psychiatric bed- and housing-capacities, alongside strengthening community care, should be considered to meet the different treatment needs of people with mental disorders. Incarceration rates in the SEAR have increased, paralleling trends in higher-income countries and reaching a similar size. The trends suggest an imbalance in development and might point towards a disproportionate risk of incarceration for people with severe mental illness. Policies aiming to reduce the ratio between imprisoned people and psychiatric beds may shift individuals back to more therapeutic settings. Future research may examine the impact of specific care components, interventions and policies to reduce the criminal justice involvement of people with severe mental disorders and to specify transitory institutional or intensive care needs.

## Introduction

The Southeast Asia Region (SEAR) of the World Health Organization (WHO) is a group of 11 countries: Bangladesh, Bhutan, Democratic People's Republic of Korea, India, Indonesia, Maldives, Myanmar, Nepal, Sri Lanka, Thailand, and Timor-Leste. The region holds over one-quarter of the world's population and has a disproportionately large burden of mental disorders.^1^ All countries in the region are low or middle-income countries (LMICs) based on the World Bank criteria.^2^ There is a large treatment gap for people with neuropsychiatric disorders,^3^ and the majority of people with mental illnesses do not receive any treatment.^4^ Most countries in the region have a national mental health policy that guides service development. In 2020, the SEAR had the lowest median mental health expenditure per capita in absolute terms (0.1 US$) and as a percentage of the total public health expenditure (0.5%).^4^ After the African Region, it had the lowest proportion of people with psychosis treated in the mental health systems (16%), the lowest prevalence of inpatient psychiatric beds, and the lowest prevalence of mental health professionals (2.8 per 100,000 population), including psychiatrists (0.4 per 100,000 population).^4^ Bed numbers in the SEAR remain substantially lower than international recommendations. A worldwide Delphi panel, including experts from the SEAR, suggested a minimum standard of 30 psychiatric beds per 100,000 population for balanced mental health services.^5^ In contrast, in the US, the need was estimated to be 50.5 per 100,000 people, in a context of highly developed outpatient care.^6^ Worldwide, need estimates for psychiatric beds have been increasingly higher than actual numbers of available beds.^7^ The functional integration of mental health into primary care, based on the availability of pharmacological and psychosocial interventions, as well as training for mental health conditions at the primary care level, was 9% in the SEAR, the lowest among all WHO world regions.^4^

Suicide rates in the region were higher than the global average (10.2 vs 9.0 per 100,000 population) in 2019, especially the female suicide rate (8.1 vs 5.4 per 100,000).^4^ In the recent decade, the use of opium and other illicit drugs in the community has been on the rise. In some countries, the use of cannabis is part of traditional or religious rituals, even though it is not legalized. Nowadays, the SEAR is particularly affected by drug use disorders^8^ and has an important role in the global trade of synthetic drugs.^9^

An increase in the prison population in several SEAR countries above the worldwide median increase in the prison population has been reported.^10^ This increase seems to be particularly pronounced in women. The female prison population has more than doubled in Asia since 2000 and makes up 7.3% of the total prison population (higher than 6.8% worldwide).^11^ Prisons in the region are typically overcrowded, and the resources are insufficient to meet basic needs and humane conditions.^12^ The prevalence of psychiatric disorders in Southeast Asia prisons was estimated to be 40–100% in prison populations, with hardly any mental health services in place.^13^ Incarceration rates have been suggested as an indicator of the institutionalization of people with mental illness, since serious mental illness and substance use disorders are highly prevalent in those populations.^14^ Many individuals experience recurrent incarcerations and psychiatric hospitalizations over time.^15^ An association between the removal of psychiatric beds and increasing incarceration rates has been observed in Latin America, another LMIC region.^16^ High rates of violent outcomes and incarceration have been described for people with the first episode of psychosis.^17^^,^^18^ Individuals with substance use disorders or traumatic brain injuries also have high rates of criminal justice involvement, especially when experiencing homelessness.^19^ When incarceration rates are high and the availability of psychiatric beds is scarce, there may be a disproportionate risk of institutionalization for people with serious mental illness in the criminal justice system. Therefore, comparing those indicators within each country and across countries may guide developments towards more balanced systems.^20^

In the SEAR, there has been very limited development of forensic psychiatric treatment capacities.^13^ Residential facilities are community-based non-hospital care facilities providing overnight residence to people with mental health conditions. The WHO Mental Health Atlas reported the availability of beds in mental health community residential facilities for only three of the 11 SEAR countries. The median prevalence was 1.4 residential beds per 100,000 population in those countries.^4^ Changes over time in the prevalence of psychiatric beds, prison populations, forensic psychiatric beds and beds in residential facilities have been reported for other world regions.^16^^,^^20^, ^21^, ^22^

The present study aimed to assess changes in indicators of psychiatric institutionalization as the prevalence of psychiatric beds, prison populations, specialized forensic beds, and beds in residential facilities in the SEAR between 1990 and 2024.

## Methods

### Data sources

An international network of researchers participated in the study. We contacted potential collaborators based on contributions to the scientific literature, snowballing, and personal contacts between May 24, 2019, and June 25, 2020. Given the high population and geographical extension of India, four collaborators from different cities participated. A standardized template was used to collect data on each indicator and year over the period between 1990 and 2024. Primary data were obtained by participating collaborators in their respective countries. When data from primary and secondary national sources were unavailable, data were retrieved from World Health Organization reports,^4^ scientific publications, and the World Prison Brief online database.^23^ Detailed data sources for each country are reported in the Supplement. The national population counts, gross national incomes in US dollars based on the Atlas method and the GINI index were retrieved from the World Bank.^2^

### Definition of indicators

Four different indicators were assessed: (1) Psychiatric beds as defined by the WHO, as any bed in hospital settings assigned to mental health treatment in psychiatric hospitals or general hospital psychiatric units (GHPU). (2) Forensic psychiatric beds included any bed reserved for the evaluation or treatment in forensic psychiatry ordered by courts of law. (3) Beds in residential or housing facilities for people with mental illness, including non-hospital community-based mental health facilities that provide overnight residence, usually serving users with relatively stable mental disorders not requiring intensive medical interventions. We excluded facilities exclusively providing treatment to people with substance use disorders or intellectual disability and generic residential facilities not intended to meet mental health needs (e.g., nursing and rest homes for older people, institutions treating neurological disorders or physical disability). (4) Prison populations were defined as all individuals confined day and night in jails or prison facilities on remand and/or sentenced.

### Statistical analysis

Rates were calculated as the number of beds or imprisoned individuals per 100,000 population. The median and interquartile range, as well as the mean and standard deviation, were calculated. The percentage changes of the prevalence for each indicator were calculated between the first and last available data points for each country, and also for the median, mean and crude total of the region. Additionally, changes in absolute numbers of psychiatric beds and prison populations were calculated for the region as a whole. We also calculated a ratio between the prevalence of prison populations and psychiatric beds in each country and the region to estimate the relationship in size between more punitive and therapeutic forms of institutionalization.^20^ Findings for SEAR were compared with those from countries forming part of the Organization for Economic Cooperation and Development (OECD) to put regional data in the context of current developments in high-income economies. The OECD is an international organization of 38 countries for which data are publicly available: numbers of psychiatric beds were retrieved from the OECD,^24^ while prison population rates were retrieved from the Institute for Crime & Justice Policy Research.^23^ Findings were presented using descriptive analyses.

### Ethics statement

All authors had access to the database. Since this study used routine service data, ethics approval was not obtained.

### Role of funding source

The funder had no role in study design, data collection, data analysis, interpretation, writing of the report or decision to submit for publication.

## Results

As of 2023, the population of the SEAR countries amounted to more than two billion people. India accounted for over 68% of the population. All countries were low- or lower-middle-income economies in 1990. Eight out of eleven countries increased the income group from the low-income to the lower middle-income category between 1990 and 2023. North Korea was the only country in the region to change from a lower middle- to a low-income economy (Table 1).

**Table 1:** Population size, income group, per capita gross national income (GNI), and Gini index indicating income inequality in Southeast Asian countries.

Country	Population 2023	Income group 1990	Income group 2023	GNI per capita 2023^a^	Gini index	
					Year	Score
Bangladesh	171,466,990	LI	LMI	2880	2022	33.4
Bhutan	786,385	LI	LMI	3600	2022	28.5
North Korea	26,418,204	LMI	LI	NA	NA	NA
India	1,438,069,596	LI	LMI	2540	2021	32.8
Indonesia	281,190,067	LI	LMI	4810	2023	36.1
Maldives	525,994	LI	UMI	11 070	2019	29.3
Myanmar	54,133,798	LI	LMI	1230	2017	30.7
Nepal	29,694,614	LI	LMI	1430	2022	30.0
Sri Lanka	22,037,000	LI	LMI	3540	2019	37.7
Thailand	71,702,435	LMI	UMI	7200	2021	34.9
Timor-Leste	1,384,286	LI	LMI	2020	2014	28.7

LI, Low-Income; LMI, Lower Middle-Income; UMI, Upper Middle-Income; HI, High Income; NA, not available.

All data were retrieved from the World Bank webpage: https://data.worldbank.org/.

a Atlas method (current US$).

From North Korea, we did not retrieve any data on psychiatric beds or imprisonment, so the country was excluded from the analyses. Primary data on the prevalence of psychiatric beds and prison population were retrieved from seven out of 10 included countries. In the three remaining countries (Indonesia, Myanmar, and Timor-Leste), we contacted at least one researcher, who either was unable to obtain the data or did not sustain contact. Data were retrieved from secondary sources in these countries. Data on specialized forensic beds were obtained from seven countries and on residential/housing facilities from three countries (Table 2).

**Table 2:** Prevalence of psychiatric beds, specialized forensic psychiatric beds, places in residential facilities for individuals with mental health problems, and prison populations in 10 Southeast Asian countries.

Country^a^	Psychiatric beds per 100,000 population				Specialized forensic psychiatric beds per 100,000 population				Beds in residential facilities per 100,000 population				Imprisoned people per 100,000 population				Imprisonment-beds ratio^c^
	Period of observation	Rate at first point	Rate at last point	Percentage change	Period of observation	Rate at first point	Rate at last point	Percentage change	Period of observation	Rate at first point	Rate at last point	Percentage change	Period of observation	Rate at first point	Rate at last point	Percentage change	Ratio at last point
Bangladesh	1992–2022	0.69	0.79	14	2007–2022	0.01	0.01	−9	2006–2022	0.92	2.30	150	1993–2022	37.70	48.86	30	62
Bhutan	1990–2024	0.00	2.54	NA	1990–2022	0.00	0.00	NA	1990–2022	0.00	0.00	NA	2011–2014	141.11	156.62	10	61
India	1990–2022	2.33	3.97	71	ND	ND	ND	NA	ND	ND	ND	NA	1993–2022	21.16	40.21	90	10
Indonesia^b^	1995–2020	4.18	5.54	33	ND	ND	ND	NA	ND	ND	ND	NA	1990–2023	22.78	97.74	329	18
Maldives	1990–2024	0.00	0.76	NA	1990–2023	0.00	0.00	NA	1990–2023	28.23	38.02	35	1990–2020	387.63	330.46	−15	435
Myanmar^b^	2001–2020	5.50	3.31	−40	ND	ND	ND	NA	ND	ND	ND	NA	1993–2020	124.03	184.39	49	56
Nepal	1990–2022	0.31	1.72	449	1990–2022	0.00	0.00	NA	ND	ND	ND	NA	1994–2022	29.47	92.71	215	54
Sri Lanka	1990–2022	9.90	8.18	−17	1990–2022	0.66	0.52	−21	1990–2022	3.72	2.91	−22	1990–2023	81.54	147.77	81	18
Thailand	1997–2024	13.58	6.42	−53	1990–2024	0.18	0.24	36	2007–2024	1.34	1.67	24	1990–2023	151.64	365.87	141	57
Timor-Leste^b^	1990–2024	0.00	0.87	NA	1990–2024	0.00	0.00	NA	ND	ND	ND	NA	2003–2021	33.79	57.76	71	67
Median rates		1.51	2.93	94		0.00	0.00	NA		1.34	2.30	72		37.70	97.74	106	56
IQR		5.09	4.07			0.09	0.12			2.80	1.24			94.56	126.62		35
Mean values		3.65	3.41	−7		0.12	0.11	−8		6.84	8.98	31		98.86	151.75	48	84
SD		4.75	2.60			0.25	0.20			12.03	16.27			118.18	121.00		125
Absolute numbers (thousands)		43	82	90		0.23	0.30	31		2.00	4.75	137		439	1335	205	
Total population (millions)^d^		1292	2070	60		237	288	22		159	192	21		1292	2070	60	
Rates for the whole region		3.32	3.95	19		NA	NA	NA		NA	NA	NA		33.88	64.46	90	16
Number of countries		10	10			7	7			5	5			10	10		10

Abbreviations: NA not applicable; ND no data; IQR Interquartile range; SD Standard deviation.

Data sources for each country are shown in the online Supplement.

a North Korea was excluded.

b No primary data were retrieved from these countries.

c Imprisonment-beds ratio indicates how many individuals are imprisoned per available psychiatric bed at the last data point.

d Population counts were retrieved from the World Bank: https://data.worldbank.org/.

### Psychiatric beds

**Fig. 1:**
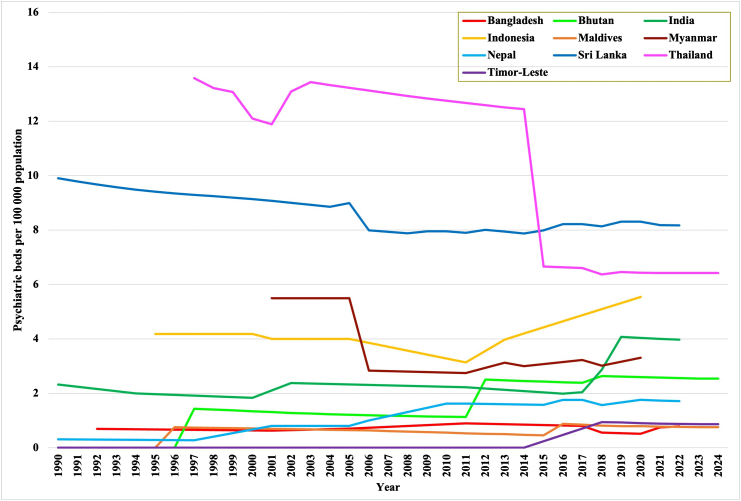
Prevalence of psychiatric beds per 100,000 population (1990–2024).

The prevalence of psychiatric beds increased when comparing the first and last data points in seven countries. Among them, the countries with the smallest population (Bhutan, Maldives, and Timor-Leste) did not have any psychiatric beds at the first data point. During the period of observation, they established psychiatric beds but were still among the countries with the lowest prevalence in the region at the last data points. The lowest prevalence was reported in Bangladesh, the Maldives, and Timor-Leste, all below one bed per 100 000 population (Fig. 1 helps visualization; data are shown in tabular form in the Supplement).

Psychiatric bed prevalence decreased in three countries (Thailand, Myanmar and Sri Lanka). The median prevalence of psychiatric beds was 1.5 at the first and 2.9 beds per 100,000 population at the last data point. The strongest increase was observed in Bhutan from 0.0 to 2.5 psychiatric beds per 100 000 population. The largest decrease in psychiatric beds was reported in Thailand from a much higher level of 13.6 per 100,000 population at the beginning of the observation period (−53%, 7.2 fewer beds per 100,000 population). A total of 42,958 psychiatric beds were calculated for the SEAR at the first data point and 81,859 at the last available data point. This implies an increase of 38,900 psychiatric beds (+91%) for the entire region. Rates for the region as a whole increased by 19%, from 3.3 to 4.0 beds per 100,000 population (Table 2).

### Prison populations

**Fig. 2:**
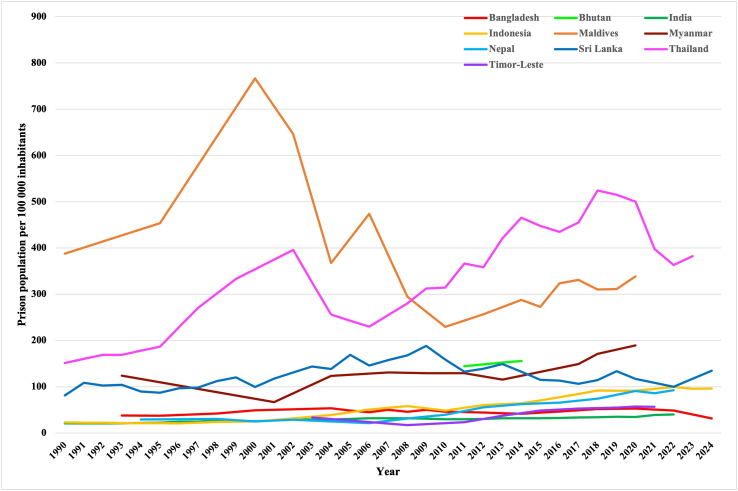
Prevalence of prison populations per 100,000 inhabitants (1990–2024).

The prevalence of imprisoned people increased in nine out of ten countries (Fig. 2 serves visualization; exact data are given in tabular form in the Supplement). The median prevalence of the regional prison population increased by 106% from 60 imprisoned individuals per 100,000 population at the first to 123 at the last data point. The percentage change ranged between the Maldives, the only country in which the prevalence decreased (−15%, 58 fewer per 100,000, but still among the highest in the region), to the highest increase in Indonesia (+329%, 75 more imprisoned individuals per 100,000).

The prison population in the region amounted to 437,620 individuals at the first data point. At the last data point, it reached 1,334,440 imprisoned people, an increase of 896,820 (205%) (Table 2).

### Specialized forensic psychiatric beds

Forensic psychiatric beds were available in seven countries (Table 2). No data were retrieved from India, Indonesia, and Myanmar. No specialized forensic beds were reported at the first and last data point in the countries with small population sizes (Bhutan, Maldives, and Timor-Leste) and in Nepal.

An unchanged total number of specialized forensic psychiatry beds was reported from Sri Lanka. However, a decrease in the prevalence (−21%, 0.1 fewer beds per 100,000 population) was observed due to population growth in the country. In Thailand, the number of forensic psychiatric beds has increased in the last decade, leading to a 36% increase in the prevalence (0.06 additional beds per 100,000 inhabitants) between the first and the last data point. A very low prevalence (0.009 per 100,000) was reported from Bangladesh at the last data point.

### Places in residential facilities

Data from only five countries were available for this indicator (Table 2). Bhutan did not have any residential/housing facilities for people with mental illnesses. Bangladesh, the Maldives and Thailand exhibited increases in the prevalence of residential places. On the other hand, in Sri Lanka there was a 22% decrease (0.76 fewer places per 100,000 inhabitants).

### Ratios between the prison populations and psychiatric beds

At the last data point, a median of 56 people were imprisoned per available psychiatric bed. The lowest ratio of 10 imprisoned individuals per available psychiatric bed was observed in India. Also, Indonesia and Sri Lanka had ratios below 20. Six of the remaining countries (Bangladesh, Bhutan, Myanmar, Nepal, Thailand, and Timor-Leste) had ratios around 60. The Maldives had a disproportionately high ratio of 435.

### Comparison with OECD countries

**Fig. 3:**
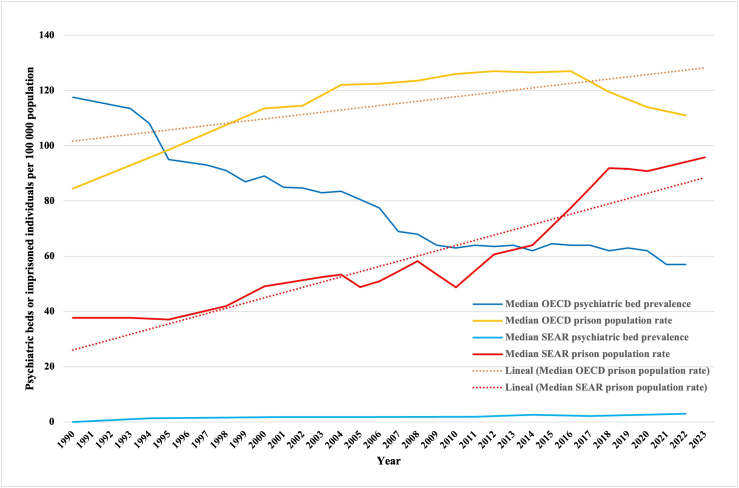
The median prevalence of psychiatric beds and prison populations in Southeast Asian countries compared with OECD countries (1990–2023).

In 2022, the median prevalence of psychiatric beds in the SEAR was about 5% of that in the OECD countries (3.0 vs 57 per 100,000 population). However, the median incarceration rate in 2023 in the SEAR (98 per 100,000 population) was similar to that in OECD countries in 2022 (111 per 100,000 inhabitants) (Fig. 3).

## Discussion

### Main findings

The prevalence of psychiatric beds increased in seven and decreased in three countries of the SEAR, while the prevalence of the prison population increased in nine out of 10 countries in the region. Data were scarce in many countries in the region, especially regarding forensic beds and residential places. Compared to the OECD countries, psychiatric beds in the SEAR are disproportionately scarce, whereas similar incarceration rates were reported.

### Interpretation

In the absence of psychiatric beds, which was the case for three countries at the beginning of the observation period, some people with acute mental illnesses may have been hospitalized in emergency departments or general medical departments, but many more of those with serious mental illness and disturbing behaviors may have been committed to detention or imprisonment. Median psychiatric bed prevalence in the SEAR was only a bit higher than in sub-Saharan Africa, but unlike in the African region, it increased in the past decades.^22^ The median psychiatric bed rates were still lower than in other LMIC regions, such as Latin America^16^ and Central Eastern Europe and Central Asia.^21^ Sri Lanka, the country with the highest prevalence of psychiatric beds in the region at the last data point, had about half of the global mean (8.2 vs 16.4 beds per 100,000),^25^ which was considered severe scarcity by worldwide standards.^5^ The prevalence of psychiatric beds in India was higher than the median of other countries in the region and increased over the past decades.^26^ In total, around 56,600 public psychiatric beds exist in the world's most populous nation.^27^ However, this amounts to only about 4 per 100,000 people, while 30 have been suggested by experts as a minimum, and 60 as an optimal number.^5^ A gradual increase in the number of GHPUs has been observed since 1982 in India when the National Mental Health Programme (NMHP) was launched,^28^ with a substantial reduction of custodial practices in psychiatric bed provision.^29^^,^^30^ It has also been described that other SEAR countries have initiated or declared intentions to develop community and social psychiatry, human rights, stigma reduction, and mental health policies,^31^^,^^32^ and to deliver mental healthcare in primary care settings.^33^ In countries with more developed mental health services, a reduction or a lower number of psychiatric beds can at least partially be compensated for by community services. However, a balance of institutionalized and community care has usually been considered in countries with much higher bed prevalence.

Beyond bed prevalence, additional factors are relevant for the use and need for psychiatric beds. In the SEAR, the length of inpatient stays was generally short (2.5% of patients staying more than one year, the lowest rate of all WHO regions).^25^ This seems related to pressure for high patient turnover due to the scarcity of beds. It could also imply that the few available beds are used rather efficiently. However, the discharge of patients to community residential facilities could be a problem, given their scarcity or absence. Thus, it may not only be difficult to access a psychiatric bed, but the quality and continuity of care may also be limited.

Although lower-middle-income countries tended to increase psychiatric bed prevalence more than upper-middle-income countries, they did so from a starting point of very low numbers (even from zero beds) and still had relatively low prevalence at the last data point when compared to other WHO regions.

However, regardless of the country's income category, there was a consistent rise in prison populations across the region. Among the three countries with decreasing psychiatric bed prevalence, the slight decline in Sri Lanka can be attributed to population growth without a corresponding increase in bed capacity. Conversely, Thailand and Myanmar actually reduced their bed numbers from a level that was comparatively high relative to other countries in the region, though still low by global standards. Whether the reduction in psychiatric beds has contributed to the marked increase in incarceration in these countries, in line with the Penrose hypothesis, may be evaluated at the national level.

High imprisonment rates, as compared to the low prevalence of psychiatric beds, may imply that people with mental illnesses and disturbing behaviors have a higher risk of criminal justice involvement rather than obtaining psychiatric hospitalization. This could indicate a societal imbalance in service development. To systematically assess this imbalance and guide future development targets for the number of psychiatric beds, we used an indicator reflecting the ratio of the prison population to the psychiatric bed numbers within the same setting. With more than 50 people in prison per psychiatric bed in most countries of the SEAR, this indicator showed higher values than in the Eastern Mediterranean region, where most countries are also LMICs.^20^ India had the lowest number on this indicator in the SEAR and may serve as an example to follow in the region for a relatively balanced development. The removal of psychiatric beds implemented in most HICs over the past decades should not be copied in LMIC regions, as most HICs had and still have substantially higher prevalences of beds. In the SEAR, a different development is needed, which will probably have to include an expansion of bed numbers. Apart from internationally established minimum numbers of psychiatric beds that may not yet be realistic for low-income settings, a minimum relationship in size of the psychiatric inpatient system compared to the correctional system could be a realistic development target. Caring for severely mentally ill in psychiatric facilities rather than in prisons may not only be more humane but also more cost-effective. If, in the future, growth in psychiatric hospitalization capacities is sustained in the SEAR, further research may evaluate whether this can contribute to stabilizing or reducing incarceration rates.

This is the first study to assess changes over three decades of indicators of institutionalization in 10 Southeast Asian countries. It presents the ratio between the prison population and psychiatric beds as an indicator to assess the balance of developments between different types of institutions. A limitation of this indicator and the study was that facilities exclusively offering treatment for patients with substance use disorders, such as therapeutic communities, were not considered. The lack of primary data sources from three of the ten countries in the region was a further limitation. Missing data points were common for several countries and indicators. We failed to obtain data on forensic beds in three countries and on residential facilities in five. Other possible explanations include that (i) national registries on those indicators do not exist in the given countries; and (ii) there are no such facilities in those countries, but this information was not reflected in an available indicator. The incompleteness of timelines resulted in different observation periods between the first and last data points in different countries. This limits the comparability of the percentage changes between them. Nevertheless, this study provides a starting point for service comparisons across jurisdictions using a common language.

Research on trends and targets for the number of beds in different psychiatric service systems is essential for the development of evidence-based public policies for improving mental health care. A major concern is the scarcity of data, and countries should improve registries with internationally comparable indicators.^21^ Over the last three decades, the SEAR seemed to have prioritized the development of prison capacities over psychiatric hospitalization, which may put people with dual severe mental health and substance use disorders at a disproportionate risk of getting institutionalized in the criminal justice system^14^ rather than being treated in the health system. Policies to improve the provision of psychiatric beds in modern general hospital settings need consideration. Future research may consider further factors beyond the static data, such as admission rates and lengths of stay in the different settings.

## Contributors

APM conceptualization, project administration, funding acquisition, supervision, and validation. SMYA, CD, AD, RKC, GSG, MK, AI, NPL, HG, and PK, provision of primary data, writing–review & editing. ERS building of research network, data curation from primary sources, and systematic search of secondary sources of information. APM, ERS, SP and CO, data analyses, writing—original draft, writing–review & editing.

## Data sharing statement

Raw data are given in the Appendix. Additional raw data can be requested from the corresponding author upon reasonable request.

## Declaration of interests

The Authors declare that there is no conflict of interest.
