# Supplementary material for: Indicators of psychiatric institutionalization in Southeast Asia between 1990 and 2024
Source: Lancet Reg Health Southeast Asia. 2025 Nov 1;42:100690. doi: 10.1016/j.lansea.2025.100690 (PMC12617761; doi:10.1016/j.lansea.2025.100690)
Supplement: Supplementary Tables [file mmc1.docx]

**Online Supplement: Indicators of psychiatric institutionalization in the South-East Asia Region between 1990 and 2024: a retrospective database study**

**Table S1.** Data sources of psychiatric bed numbers and prison populations in countries from the Southeast Asian region SEAR.

|  | **WHO Mental Health^1^ Atlas^1^** | **World Prison Brief^2^** | **Additional sources on bed availability or prison population** |
| --- | --- | --- | --- |
| **Bangladesh** | X | X | - OECD/WHO (2020), *Health at a Glance: Asia/Pacific 2020: Measuring Progress Towards Universal Health Coverage*, OECD Publishing, Paris, <https://doi.org/10.1787/26b007cd-en>.  - WHO-AIMS Report on Mental Health System in Bangladesh, WHO and Ministry of Health & Family Welfare, Dhaka, Bangladesh, 2006. |
| **Bhutan** | X | X | - WHO-AIMS Report on Mental Health System in Bhutan, WHO and Ministry of Health, Thimphu, Bhutan, 2007. |
| **India** | X | X | - [Bhugra,](https://www.routledgehandbooks.com/author/Dinesh_Bhugra) D; Tse, S; Ng R; Takei, N, ["Routledge Handbook of Psychiatry in Asia"](https://www.routledgehandbooks.com/doi/10.4324/9781315884622)(Abingdon: Routledge, 20 ago 2015), accessed 13 nov 2023, Routledge Handbooks Online.  - Math, Suresh Bada; Gowda, Guru S.; Basavaraju, Vinay; Manjunatha, Narayana; Kumar, Channaveerachari Naveen; Enara, Arun; Gowda, Mahesh^1^; Thirthalli, Jagadisha. Cost estimation for the implementation of the Mental Healthcare Act 2017. Indian Journal of Psychiatry 61(Suppl 4):p S650-S659, April 2019.  - Nagaraja, D. & Murthy, Pratima. Mental Health Care and Human Rights. National Human Rights Commission, New Delhi and National Institute of Mental Health and Neuro Sciences, Bangalore. 1st Ed, 2008. ISBN- 978-81-9044117-5.  - Krishnamurthy K, Venugopal D, Alimchandani AK. Mental hospitals in India. Indian J Psychiatry. 2000 Apr;42(2):125-32.  - National Mental Health Program: A Progress Report (1982-1990) *Directorate General of Health Services, Ministry of Health and Family Welfare.*1990.  - OECD/WHO (2018), *Health at a Glance: Asia/Pacific 2018: Measuring Progress towards Universal Health Coverage*, OECD Publishing, Paris. |
| **Indonesia** | X | X | Shinfuku N. Mental health services in Asia: international perspective and challenge for the coming years. Psychiatry Clin Neurosci. 1998 Jun;52(3):269-74.  - The Association of Southeast Asian Nations (ASEAN). (2016). *ASEAN Mental Healt Systems*. Retrieved from <https://asean.org/wp-content/uploads/2016/12/55.-December-2016-ASEAN-Mental-Health-System.pdf> |
| **Maldives** | X | X | - Mohamed A. Resuscitating the National Mental Health Policy in the Maldives. *Australasian Psychiatry*. 2015;23 (6_suppl):26-28.  - WHO-AIMS Report on Mental Health System in Maldives, WHO and Ministry of Health, Male, Maldives, 2006. |
| **Myanmar** | X | X | - WHO-AIMS Report on Mental Health System in Myanmar, WHO, Yangon, and Ministry of Health, Nay Pyi Taw, Myanmar, 2006  - [Bhugra D](https://www.routledgehandbooks.com/author/Dinesh_Bhugra); [Tse](https://www.routledgehandbooks.com/author/Samson_Tse) S; Ng R; [Takei](https://www.routledgehandbooks.com/author/Nori_Takei) N. ["Routledge Handbook of Psychiatry in Asia"](https://www.routledgehandbooks.com/doi/10.4324/9781315884622)First Edition, 2016. |
| **Nepal** | X | X | - WHO-AIMS Report on Mental Health System in Nepal, WHO and Ministry of Health, Kathmandu, Nepal, 2006.  - Luitel NP, Jordans MJ, Adhikari A, Upadhaya N, Hanlon C, Lund C, Komproe IH. Mental health care in Nepal: current situation and challenges for development of a district mental health care plan. Confl Health. 2015 Feb 6;9:3.  - Tausig M, Subedi S. The modern mental health system in Nepal: organizational persistence in the absence of legitimating myths. Soc Sci Med. 1997 Aug;45(3):441-7. |
| **Sri Lanka** |  |  | - Direct contact with those in charge of each psychiatric unit and synthesis by HG.  - Department of Prisons of Sri Lanka, <http://prisons.gov.lk/web/en/statistics-information-en/> |
| **Thailand** | X |  | - Department of Mental Health, Ministry of Public Health  - Total prison population data from Department of Corrections: <http://www.correct.go.th/> |
| **Timor-Leste** | X | X | - Hawkins Z. A decade of mental health services in Timor-Leste. Int Psychiatry. 2010 Jan 1;7(1):11-13. PMID: 31508019; PMCID: PMC6734948.  - Teresa Hall, Ritsuko Kakuma, Lisa Palmer, João Martins, Harry Minas, Michelle Kermode, Are people-centred mental health services acceptable and feasible in Timor-Leste? A qualitative study, Health Policy and Planning, Volume 34, Issue Supplement_2, November 2019, Pages ii93–ii103. |
| 1 World Health Organization. Mental Health Atlas 2020. 2021.  2 Institute for Crime & Justice Policy Research. World Prison Brief. 2025; Retrieved Apr 22, 2025 from: <https://www.prisonstudies.org/>. | | | |

| **Country** | **Prevalence per**  **100 000 population** | **1990** | **1991** | **1992** | **1993** | **1994** | **1995** | **1996** | **1997** | **1998** | **1999** | **2000** | **2001** | **2002** | **2003** | **2004** | **2005** | **2006** | **2007** | **2008** | **2009** | **2010** | **2011** | **2012** | **2013** | **2014** | **2015** | **2016** | **2017** | **2018** | **2019** | **2020** | **2021** | **2022** | **2023** | **2024** |
| --- | --- | --- | --- | --- | --- | --- | --- | --- | --- | --- | --- | --- | --- | --- | --- | --- | --- | --- | --- | --- | --- | --- | --- | --- | --- | --- | --- | --- | --- | --- | --- | --- | --- | --- | --- | --- |
| **Bangladesh** | Psychiatric beds |  |  | 0.7 |  |  |  |  |  |  |  |  | 0.6 |  |  |  | 0.7 |  |  |  |  |  | 0.9 |  |  |  |  |  | 0.8 | 0.6 |  | 0.5 | 0.7 | 0.8 |  |  |
|  | Prison population |  |  |  | 35.2 |  | 34.9 |  |  | 39.7 |  | 46.6 |  | 49.1 |  | 51.2 | 46.9 | 43.0 | 48.4 | 44.3 | 48.5 | 44.4 | 44.4 | 43.4 |  | 40.7 |  | 45.5 |  | 51.0 |  | 53.0 |  | 48.9 |  | 31.4 |
| **Bhutan** | Psychiatric beds | 0.0 | 0.0 | 0.0 | 0.0 | 0.0 | 0.0 | 0.0 | 1.4 | 1.4 | 1.4 | 1.3 | 1.3 | 1.3 | 1.3 | 1.2 | 1.2 | 1.2 | 1.2 | 1.2 | 1.2 | 1.1 | 1.1 | 2.5 | 2.5 | 2.5 | 2.4 | 2.4 | 2.4 | 2.6 | 2.6 | 2.6 | 2.6 | 2.6 | 2.5 | 2.5 |
|  | Prison population |  |  |  |  |  |  |  |  |  |  |  |  |  |  |  |  |  |  |  |  |  | 141.1 |  | 149.5 | 152.6 |  |  |  |  |  |  |  |  |  |  |
| **India** | Psychiatric beds | 2.3 |  |  |  | 2.0 |  |  |  |  |  | 1.8 |  | 2.4 |  |  | 2.5 |  |  |  |  |  | 2.2 |  |  |  |  | 2.0 | 2.0 | 2.9 | 4.1 |  | 4.0 | 4.0 |  |  |
|  | Prison population | 21.2 |  |  | 21.3 |  | 22.5 |  |  | 27.1 |  | 25.7 |  | 29.4 |  | 29.2 |  | 31.8 |  | 31.9 |  | 29.7 | 29.6 | 30.1 | 31.8 | 31.9 | 31.6 | 32.2 |  | 33.9 | 34.5 |  | 39.2 | 40.2 |  |  |
| **Indonesia** | Psychiatric beds |  |  |  |  |  | 4.2 |  |  |  |  |  | 4.0 |  |  |  | 4.0 |  |  |  |  |  | 3.1 |  | 3.9 | 4.2 |  |  |  |  |  | 5.5 |  | 5.5 |  |  |
|  | Prison population | 22.5 |  |  | 21.3 |  |  | 20.4 |  | 23.3 |  | 24.7 |  | 30.6 |  | 38.3 |  | 49.9 |  | 57.1 |  | 47.9 |  | 59.6 |  | 63.1 |  | 76.6 |  | 91.1 |  | 91.4 |  | 99.6 | 95.8 | 96.4 |
| **Maldives** | Psychiatric beds | 0.0 | 0.0 | 0.0 | 0.0 | 0.0 | 0.0 | 0.8 | 0.7 | 0.7 | 0.7 | 0.7 | 0.7 | 0.7 | 0.7 | 0.7 | 0.7 | 0.6 | 0.6 | 0.6 | 0.6 | 0.6 | 0.5 | 0.5 | 0.5 | 0.5 | 0.5 | 0.9 | 0.9 | 0.8 | 0.8 | 0.8 | 0.8 | 0.8 | 0.8 | 0.8 |
|  | Prison population | 387.6 |  |  |  |  | 453.5 |  |  |  |  | 766.7 |  | 646.3 |  | 367.4 |  | 473.6 |  | 294.8 |  | 229.6 |  | 256.5 |  | 290.1 | 277.7 | 331.4 | 341.5 | 321.1 | 321.9 | 338.6 |  |  |  |  |
| **Myanmar** | Psychiatric beds |  |  |  |  |  |  |  |  |  |  |  | 5.5 |  |  |  | 5.5 | 2.8 |  |  |  |  | 2.7 |  | 3.1 | 3.0 |  |  | 3.2 | 3.0 |  | 3.3 |  |  |  |  |
|  | Prison population |  |  |  | 124.0 |  |  |  |  |  |  |  | 67.0 |  |  | 123.5 |  |  | 131.1 |  | 129.5 |  | 129.4 |  | 115.7 |  |  |  | 149.2 | 171.3 |  | 184.4 |  |  |  |  |
| **Nepal** | Psychiatric beds | 0.3 |  |  |  |  |  |  | 0.3 |  |  |  | 0.8 |  |  |  | 0.8 | 1.0 |  |  |  | 1.6 | 1.6 |  |  |  | 1.6 | 1.8 | 1.8 | 1.6 |  | 1.8 | 1.7 | 1.7 |  |  |
|  | Prison population |  |  |  |  | 28.5 |  |  |  | 29.6 |  | 24.4 |  | 28.2 |  | 24.6 |  | 21.0 |  | 31.1 |  | 39.3 |  | 54.2 |  | 60.8 |  | 64.3 |  | 75.8 |  | 90.8 | 86.2 | 92.7 |  |  |
| **Sri Lanka** | Psychiatric beds | 10.5 | 10.4 | 10.3 | 10.1 | 9.9 | 9.8 | 9.6 | 9.4 | 9.2 | 9.1 | 8.9 | 8.8 | 8.7 | 8.6 | 8.5 | 8.7 | 7.7 | 7.7 | 7.6 | 7.7 | 7.7 | 7.7 | 7.7 | 7.9 | 7.9 | 8.0 | 8.2 | 8.2 | 8.1 | 8.3 | 9.8 | 8.2 | 8.2 |  |  |
|  | Prison population | 86.4 | 115.4 | 109.0 | 109.5 | 94.0 | 90.5 | 99.2 | 99.4 | 111.9 | 118.6 | 97.0 | 113.5 | 126.3 | 138.7 | 133.9 | 163.4 | 141.2 | 152.8 | 162.7 | 182.5 | 153.9 | 128.6 | 134.1 | 149.4 | 132.8 | 114.9 | 113.4 | 106.5 | 114.7 | 133.8 | 90.6 | 65.7 | 136.7 | 148.6 |  |
| **Thailand** | Psychiatric beds |  |  |  |  |  |  |  | 13.6 | 13.3 | 13.1 | 13.6 | 14.0 | 13.1 | 13.4 | 13.3 | 13.2 | 13.1 | 13.0 | 12.9 | 12.8 | 12.7 | 12.6 | 12.5 | 12.5 | 12.4 | 6.6 | 6.6 | 6.6 | 6.3 | 6.4 | 6.4 | 6.4 | 6.4 | 6.4 | 6.4 |
|  | Prison population | 153.0 |  |  |  |  | 187.9 |  |  |  |  | 354.6 |  | 395.3 |  | 255.4 |  | 229.3 |  | 279.2 | 311.1 | 313.1 | 364.9 | 356.8 | 419.1 | 463.8 | 446.1 | 433.2 | 453.4 | 522.6 | 513.4 | 500.2 | 397.7 | 363.0 | 382.5 |  |
| **Timor-Leste** | Psychiatric beds | 0.0 |  |  |  |  |  |  |  |  |  |  |  |  |  |  |  |  |  |  |  | 0.0 | 0.0 |  |  | 0.0 |  |  | 0.0 | 0.9 | 0.9 | 0.9 | 0.9 | 0.9 | 0.9 | 0.9 |
|  | Prison population |  |  |  |  |  |  |  |  |  |  |  |  |  | 34.9 |  |  |  |  | 17.3 |  |  | 203.6 |  | 37.0 |  | 48.2 |  | 52.6 |  | 55.0 | 57.1 | 56.5 |  |  |  |

**Table S2.** Prevalence of psychiatric beds and imprisoned people per 100 000 population between 1990 and 2024 in ten SEAR countries.
